# Rapid Screen of the Color and Water Content of Fresh-Cut Potato Tuber Slices Using Hyperspectral Imaging Coupled with Multivariate Analysis

**DOI:** 10.3390/foods9010094

**Published:** 2020-01-16

**Authors:** Qinlin Xiao, Xiulin Bai, Yong He

**Affiliations:** 1 College of Biosystems Engineering and Food Science, Zhejiang University, Hangzhou 310058, China; qinlxiao@zju.edu.cn (Q.X.); xlbai@zju.edu.cn (X.B.); 2 Key Laboratory of Spectroscopy Sensing, Ministry of Agriculture and Rural Affairs, Hangzhou 310058, China

**Keywords:** hyperspectral imaging, fresh-cut potato tuber slices, browning, water content, color index

## Abstract

Color index and water content are important indicators for evaluating the quality of fresh-cut potato tuber slices. In this study, hyperspectral imaging combined with multivariate analysis was used to detect the color parameters (L*, a*, b*, Browning index (BI), L*/b*) and water content of fresh-cut potato tuber slices. The successive projections algorithm (SPA) and competitive adaptive reweighted sampling (CARS) were used to extract characteristic wavelengths, partial least squares (PLS) and least squares support vector machine (LS-SVM) were utilized to establish regression models. For color prediction, R^2^_c_, R^2^_p_ and RPD of all the LSSVM models established for the five color indicators L*, a*, b*, BI, L*/b* were exceeding 0.90, 0.84 and 2.1, respectively. For water content prediction, R^2^_c_, R^2^_p_, and RPD of the LSSVM models were over 0.80, 0.77 and 1.9, respectively. LS-SVM model based on full spectra was used to reappear the spatial distribution of color and water content in fresh-cut potato tuber slices by pseudo-color imaging since it performed best in most cases. The results illustrated that hyperspectral imaging could be an effective method for color and water content prediction, which could provide solid theoretical basis for subsequent grading and processing of fresh-cut potato tuber slices.

## 1. Introduction

Potato is one of the largest food crop throughout the world. Potato is rich in carbohydrates, potassium, vitamin C and vitamin B6, making it an excellent food source [1,2,3]. With the demands by consumers, washed and sliced potato tubers are widely welcomed due to their convenience in processing. The color and appearance characteristics of potato tuber slices directly affect the acceptability of consumers. However, the integrity of potato tuber slices is destroyed due to the cutting process, and the polyphenol oxidase is directly exposed to the air. The oxygen environment accelerates the oxidation of phenolic compounds to form quinones-based polymers, which eventually lead to pigment formation. This series of reactions are enzymatic browning [4]. Browning not only deepens the color of potato tuber slices, but also impacts their sensory quality. Moreover, browning directly leads to a decrease in consumers’ desire to purchase, causes nutrition loss, shortens shelf life [5]. Fresh-cut potato tuber slices are also prone to water loss at room temperature condition, which accelerates the decline of sensory quality. Therefore, the color and water content largely reflect the freshness of fresh-cut potato tuber slices. Rapid detection of color and water content can help determine the quality of fresh-cut potato tuber slices and provide a theoretical basis for quality monitoring and food grading.

Generally, water content is determined by drying method. Its value can be calculated from initial and constant weight of the sample before and after being dried. The entire process takes a long time and causes irreversible damage to the sample, making it hard achieving large-scale detection quickly. As for color inspection, chromatic meter is an effective tool for characterizing the color of foods according to the difference between the tested sample and the standard whiteboard in terms of hue, lightness and chroma. However, existing commercial chromatic meters cannot perform global analysis of the entire surface due to the fact that they measure only a few square centimeters at a time. Therefore, to obtain a set of data that can effectively reflect the color of the whole sample, multiple measurements are necessary and the average value is regarded as the representative data. Though this technique obtains objective data, cumbersome operation limits its widespread application. Imaging technique, which is nondestructive and convenient to operate, provides a new possibility for agricultural product quality evaluation. Among these imaging techniques, computer vision is widely used, especially in moisture and color detection [6,7,8]. However, computer vision only obtains the two-dimensional information of samples in visible bands. As a combination of spectral technique and imaging technique, hyperspectral imaging (HSI) not only obtains one-dimensional spectral information of samples both in visible region and near infrared region, but also captures two-dimensional spatial information [9]. Compared with the traditional methods, HSI is easy to operate, which contributes to analyze the content and distribution of multiple components at the same time, making the whole detection process more efficient [10]. HSI has been used in various studies to detect moisture and color in recent years [11,12,13,14]. These studies all showed the feasibility of characterizing water content and chromaticity by HSI. As for the quality assessment of potatoes, HSI has been used in the following studies: prediction of pigment content in purple-fleshed sweet potato tuber slices [15], sugar content detection [16], determination of starch content [17], prediction of starch, soluble sugars and amino acids [18], identification of sliced organic potatoes [19], prediction of sprouting potato eyes [20], evaluation of optimal cooking time for boiled potatoes [21], classification of defective potatoes [22], as well as detection of blackspot [23]. Moreover, Sun et al. [24] utilized HSI to predict the moisture content and freezable water content of purple-fleshed sweet potato slices during drying process. Arnold and DeBiasio [25] investigated the potential of using near-infrared imaging spectroscopy to identify the browning areas in French fries. Amjad et al. [26] used HSI to characterize the water content and chromaticity of potato tuber slices during the convective hot air drying process. Moscetti et al. [27] further studied the changes of water content and browning of potato tuber slices during hot-air drying by HSI, which both confirmed the feasibility of applying HSI for determination of water content and color in potato tuber slices. However, despite these miscellaneous applications, only few studies have focused on rapid visualization of color and water content of fresh-cut potato chips simultaneously under normal temperature storage conditions using HSI.

Therefore, this study was performed to rapid screen of color and water content of fresh-cut potato tuber slices using HSI coupled with multivariate analysis, so as to provide a certain reference for potato quality evaluation. The specific objectives are: (1) obtain hyperspectral images of fresh-cut potato tuber slices at the spectral range of 380–1030 nm; (2) identify the characteristic wavelengths using successive projections algorithm (SPA) and competitive adaptive reweighted sampling (CARS); (3) compare the predictive ability of partial least squares (PLS) and least squares support vector machine (LS-SVM) respectively and compare their performances based on the full spectra and the characteristic wavelengths; (4) develop visualization map of color and water content distribution of fresh-cut potato tuber slices.

## 2. Materials and Methods

### 2.1. Sample Preparation

The experiment was carried out using commercial potatoes (cultivar: Holland fifteen, geographical origins: Weifang, Shandong Province, China) which were procured from the same supermarket at different times. The potato tubers were cut into slices with a thickness of 3 mm along the long axis using a stainless steel slicer. The potato tuber slices were placed in a plastic box wrapped with cling film to protect the slices from microbial contamination. Then the packaged potato tuber slices were placed at room temperature (25 °C) for a certain placement time (0 h, 1 h, 2 h, 4 h, 8 h, 12 h, 24 h, 36 h) to simulate the shelf environment. After storage, hyperspectral image acquisition, chromaticity measurement and water content measurement of the potato tuber slices were conducted.

In this experiment, 30 potato tuber slices from six different potatoes were set for each time gradient, resulting in 240 samples in a total. The purpose of setting time gradient is to obtain representative samples for color parameters and water content measurement. Due to the large individual differences between the potatoes, the color and water content of samples set under different time gradients did not change continuously. The changes of color and water content over time are not considered here.

### 2.2. Color Measurement and Water Content Measurement

After hyperspectral image acquisition, the potato tuber slices were arranged to perform color measurement and water content measurement. A Chroma-Meter (CR-400, Konica Minolta Optics Inc., Tokyo, Japan) with the CIELAB color system was used to measure the color of potato tuber slices. The measurement was conducted ten times at different positions of each sample and three readings of luminance (L*), redness (a*) and yellowness (b*) were recorded. The average of these ten measurements was selected as the chromaticity value of the sample. In addition, we used the average value of L*, a* and b* to calculate the browning index (BI) [28] and luminance/yellowness ratio (L*/b* ratio) [27].

(1) $$\mathrm{BI}=\frac{100\times \left(x-0.31\right)}{0.172},\;x=\frac{a^{*}+1.75\times L^{*}}{5.645\times L^{*}+a^{*}-3.012\times b^{*}}$$

Water content measurement was carried out by using a convective hot-air oven (BA0-150A, STIK Ltd., Shanghai, China) and an electronic balance (BT125S, Sartorius Ltd., Beijing, China) with a precision of 0.1 mg. The potato tuber slices were dried at 70 Celsius degrees (°C) in the oven and weighed with an electronic balance until reached a constant weight. Three replications were performed for each measurement and took the average as the true value. We calculated water content (*WC*) by the following formula,

(2) $$WC=\frac{w_{c}-w_{d}}{w_{c}}$$

In the above formula, *WC* represents the water content (g water g wet matter^−1^), *Wc* and *Wd* represent the weight of potato tuber slices before and after drying, respectively.

To build calibration models for color indexes and water content prediction, the potato tuber slices were sorted according to the increasing order of the measured values of each parameter after outlier removal. The middle sample of every three samples was selected into the prediction set, and the remaining two samples were selected into the calibration set. Therefore, the number of samples in the calibration and prediction set was 156 and 78 for each parameter, respectively.

### 2.3. Hyperspectral Image Acquisition

#### 2.3.1. Hyperspectral Imaging System

Hyperspectral images acquisition was conducted by a laboratory-based line-scanning hyperspectral imaging system. The system consists of an imaging spectrograph (ImSpector V10E; Spectral Imaging Ltd., Oulu, Finland), a 672 × 512 (spatial × spectral) CCD camera (C8484-05, Hamamatsu, Hamamatsu City, Japan) with a camera lens (OLES23; Spectral Imaging Ltd., Oulu, Finland), an illumination unit of two 150 W tungsten halogen lamps (3900e Lightsource; Illumination Technologies Inc.; West Elbridge, NY, USA), a displacement platform driven by a stepper motor (Isuzu Optics Corp., Taiwan, China) and a computer equipped with a matched data acquisition software (Xenics N17E, Isuzu Optics Corp.). The data acquisition was carried out in visible and near-infrared region (Vis-NIR, 380–1030 nm) and reflectance mode was applied. Since the entire sample area was defined as the region of interest (ROI) and all the pixels in the image has their corresponding spectra, the spectra of all the pixels in the ROI were obtained at once measurement.

#### 2.3.2. Imaging Acquisition and Calibration

The potato tuber slices were placed on a black plate on the mobile platform to move into the camera field of view for hyperspectral image acquisition. In order to obtain a clear and non-deformed hyperspectral image, the distance between the camera lens and the sampling plane was set to 14.5 cm, the plate moving speed was 0.85 mm/s, and the camera exposure time was set to 0.14 s.

In view of the fact that the deviations produced by the detector itself and the interference caused by the illumination, recording the white reference image (*I_white_*) and the dark reference image (*I_dark_*) under the same experimental conditions to correct raw images is necessary. The white and black reference images were obtained from a collection of white Teflon bar images (about 100% reflectance) and image which was captured in condition of turning off the light source and covering the camera lens completely (about 0% reflectance). The calibrated image (*I_c_*) was calculated as follow:

(3) $$I_{c}=\frac{I_{raw}-I_{dark}}{I_{write}-I_{dark}}$$

In the above equation, *I_c_*, *I_raw_*, *I_white_* and *I_dark_* are the calibrated hyperspectral image, raw hyperspectral image, the white and dark reference images, respectively.

#### 2.3.3. Image Preprocessing and Spectral Extraction

The segmentation between potato tuber slices and background is an important start of extracting spectral data. Each piece of potato tuber slice was defined as the region of interest (ROI), and the ROI was firstly segmented using ENVI software (ENVI 5.1, ITT Visual Information Solutions, Boulder, CO, USA). As the maximum difference between the background and the ROI area was observed at 590 nm. The grayscale image of this wavelength was used to create a mask with a threshold of 0.1, which was applied to perform simple threshold segmentation on whole hyperspectral images to remove the background information. Then, spectral extraction was carried out for the ROI area. Removing the head-to-tail spectra with high noise levels, the spectral range for further analysis was set as 477–947 nm (370 bands). The pixel-wise spectra in each ROI were preprocessed using wavelet transform of Daubechies 9 with decomposition scale of 3 and area normalization. The spectra extraction procedure was implemented in MATLAB R2015b (The MathWorks, Matick, MA, USA).

### 2.4. Data Analysis

#### 2.4.1. Regression Models

Regression is a data analysis method that attempts to find out the correlation between two sets of random variables. Through regression analysis, the correlation degree between the independent variable *X* and the response variable *Y* can be found, so as to realize the prediction of the *Y* value by analyzing the *X* value of new samples by establishment of the regression model *f*(*x*). Therefore, the regression model based on calibration set can explain the relationship between the spectral (*X*-variable) and the measured characteristic value (*Y*-variable), the response characteristic value of the prediction set can be predicted by taking the corresponding spectra as the input of the model. In this study, partial least squares (PLS) and least squares-support vector machine (LS-SVM) regression models were built using the average spectra of all pixels in the sample area (*X*-variable) and the corresponding chromaticity values (L*, a*, b*, BI, L*/b*) and water content (*Y*-variable), respectively.

PLS is a commonly used linear regression analysis method, which combines the functions of multiple regression analysis and principal component analysis. PLS can be used for modeling analysis under the condition that the independent variables have relatively serious multi-collinearity, and it is applicable to the case that the number of samples is far less than the number of variables [29]. PLS can perform data processing on *X*-variables and *Y*-variables at the same time. The main information in the two sets of matrices is extracted by optimizing the covariance of *X*-variables and *Y*-variables [30]. The establishment of the PLS regression model and ten-fold cross-validation were performed in the Unscrambler X 10.1 (Camo AS, Oslo, Norway).

LS-SVM is an extension of the traditional SVM method. Different from the traditional SVM model, LS-SVM mainly adopts the least square linear system as the loss function, which simplifies the complexity of calculation. In addition, LS-SVM is able to analyze linear and nonlinear multivariable problems accurately. In this study, radial basis function (RBF) was selected as the kernel function to better deal with the nonlinear relationship between spectral and index values [30]. The setting of regularization parameters gamma (γ) and RBF kernel parameters (σ^2^) are very important for the establishment of LS-SVM model with high regression accuracy. In order to achieve the best performance of the LS-SVM regression model, the regularization parameters gamma (γ) and RBF kernel parameters (σ^2^) of the kernel function need to be optimized. In this study, through 1000 repetitions of modeling optimization, the optimal values of γ and σ^2^ were selected as the parameter combination for constructing LS-SVM model and calculating the minimum root mean square error (RMSECV) for cross-validation. In the process of validation, ten-fold cross-validation was adopted. The establishment of LS-SVM model was followed the method in study [31].

#### 2.4.2. Wavelength Selection

Spectral information in continuous bands is obtained by HSI. Each pixel in the hyperspectral image has a corresponding spectral value at each band, and an image contains hundreds or thousands of pixels, which makes it cover a large amount of data. However, massive data not only provides useful information for regression modeling, but also contains redundant, collinear information and noise. These parts of information will reduce the accuracy of the model and slow down the speed of subsequent modeling and analysis. Therefore, wavelength selection is essential to pick up the wavelengths with the minimum collinearity, the least redundancy and the main effective information for multiple regression modeling. In this study, successive projections algorithm (SPA) and competitive adaptive reweighted sampling (CARS) were used to select the characteristic wavelengths.

SPA is an effective forward variable selection method. It can extract effective information from highly collinear variables and minimize the influence of collinearity [32]. Thereby, the characteristic wavelengths are extracted from the full bands, most of the redundant information in the original spectral matrix is eliminated and the modeling conditions can be improved. The basic principle of SPA is to simply project a set of wavelength subsets in the vector space and select the wavelength subset with the least redundancy. In this study, the number of selected variables was selected at the range 5–30.

CARS is a variable selection method that combines adaptive reweighted sampling (ARS) and PLS. The main principle of the CARS algorithm is to preserve the wavelength with larger absolute weights of the regression coefficients in the PLS model as a new subset by adaptive weighted sampling, remove the wavelength with smaller weights, and then build the PLS model based on the new subset [33]. After multiple cyclic iteration, the wavelength subset with the smallest RMSECV in PLS models was selected.

#### 2.4.3. Model Evaluation

Evaluating the performance of the constructed model with appropriate indicators is a crucial part of assessing its practical application ability. The reliability and robustness of the model can be assessed by the model performance evaluation. In this study, the coefficients of determination (R^2^) in calibration, validation and prediction set (R^2^_c_, R^2^_cv_ and R^2^_p_, respectively), root mean square error (RMSE) of calibration, validation and prediction set (RMSEC, RMSECV and RMSEP) and residual prediction deviation (RPD) were used to evaluate model performance. The closer R^2^ of the model is to 1, the closer RMSE is to 0, the higher the value of RPD, indicating that the variables are more capable to explain the response value, which also means that the performance of the model is better. According to the reference [34], the value of R^2^ between 0.61 and 0.8 demonstrates that the model is capable for prediction. An R^2^ of 0.81–0.9 indicates that the model shows good performance, and its value above 0.9 manifests the prediction ability of the model is excellent. The smaller the RMSEs, suggesting the better the model’s fitting ability. RPD is the ratio of standard deviation (SD) of the prediction set to RMSEP. Its value below 2.0 indicates the model is incapable to perform quantitative prediction, a value of 2.0–2.5 means that the model can be applied to prediction and a value above 2.5 proves the prediction ability of the model is good [34]. R^2^, RMSE, and RPD of the calibration, validation, and prediction set were calculated using the following equations.

(4) $$R^{2}=1-\frac{\sum_{i=1}^{n}{\left(\hat{y_{i}}-y_{i}\right)}^{2}}{\sum_{i=1}^{n}{\left(\hat{y_{i}}-y_{mean}\right)}^{2}}$$

(5) $$\mathrm{RMSE}=\sqrt{\frac{\sum_{i=1}^{n}{\left(\hat{y_{i}}-y_{i}\right)}^{2}}{n}}$$

(6) $$\mathrm{RPD}=\frac{SD}{RMSEP}$$

where *n* is the number of samples in corresponding sample set, $\hat{y_{i}}$ is the predicted value of the ith sample obtained by the regression model, $y_{i}$ is the value of the ith sample measured by the reference method, and $y_{mean}$ is the mean value of the measured value.

## 3. Results and Discussion

### 3.1. Color Parameters and Water Content Distribution

Table 1 shows detailed statistical data on the color index and water content of all fresh-cut potato tuber slices. Among all the color indicators, range of L* value covered 43.794–64.738, the larger the L* value indicated the higher the brightness of potato tuber slices. The range of a* value was −3.096 to 2.050, and the scale of a* value suggested the degree of the red or green of the sample. Negative values indicated that the potato tuber slices were greenish, positive values indicated that the potato tuber slices were redder conversely. The b* value revealed the yellowish or blue level of the sample. The range of b* was 11.247–20.681. The larger the b* value, the more obvious the yellow color of the sample. The results were similar to the existing research [27], within the normal range. Since the color change was mainly caused by browning reaction on the surface of potato tuber slices, the browning index was also used to evaluate the surface color in this study. The greater the browning index, the more pronounced the browning of the surface. In the research of Moscetti et al. [27], quality inspection of potato was carried out during hot air drying process, L*/b* was also used as an indicator to evaluate the color of potato tuber slices. L*/b* decreased as the color deepened. The water content of all fresh-cut potato tuber slices ranged from 0.753 to 0.879, covering the water content of fresh-cut potato tuber slices after being placed at 25 °C for 0h to 36 h. It was worth noting that the high standard deviation of each indicator indicated the high difference in fresh-cut potato slice samples. Reasonable chemical value distribution facilitated the establishment of a highly robust model. In addition, the range of chemical values of calibration set included that of prediction set, and the mean value and standard deviation of chemical values did not show significant deviation between the calibration set and prediction set.

### 3.2. Spectral Profiles

**Figure 1:**
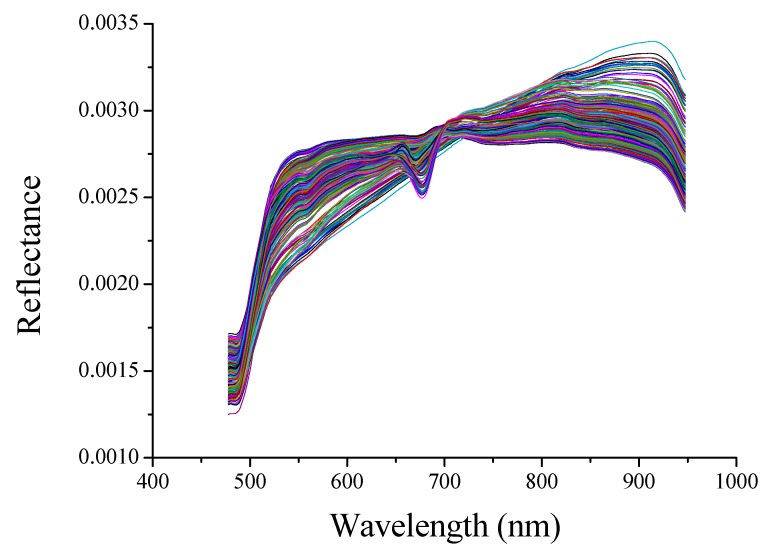
Reflectance spectra of fresh-cut potato tuber slices.

Figure 1 shows the preprocessed reflectance spectra of all fresh-cut potato tuber slices. Since the noise was significant at the head and tail of the spectra, only the spectra in the range 477–948 nm was considered. The spectral trends of all samples were basically the same, but the reflectance in the range of 650–750 nm showed some differences. On the one hand, the reason might be that potato tuber slices were taken from multiple potatoes, and the individual differences between samples were quite large. On the other hand, it might be caused by changes in the water content and surface color of fresh-cut potato tuber slices during placement process. A valley (678 nm) and a peak (705 nm) were observed in some spectral curves. From the perspective of visible light, 678 nm and 705nm were associated with redness. Moreover, the wavelengths near 678 nm and 705 nm were also designated as the fourth overtone of C–H stretch [35]. All the wavelengths contained a mass of complex information about the color and composition of the samples. Multivariate analysis method was applied to explore the relationship between spectra and color indexes (L*, a*, b*, BI, L*/b*) and water content of fresh-cut potato tuber slices.

### 3.3. Regression Models

#### 3.3.1. Regression Models for Color Prediction

In this study, PLS and LS-SVM were used to establish prediction models of the color indexes L*, a*, b*, BI and L*/b* respectively. SPA and CARS were applied to select the characteristic wavelengths. Based on above methods, Prediction models were built based on the full spectra and the selected characteristic wavelengths (shown in Table S1 in Supplementary file) respectively. Table 2 shows the prediction results of L*, a*, b*, BI, L*/b* values. In terms of the prediction of L*, a*, b*, BI, L*/b*, PLS and LS-SVM models based on full spectra and characteristic wavelengths all obtained good performance.

For the prediction of L*, the performance of PLS models was less than satisfactory, with R^2^_p_ in the range of 0.736-0.801 and RPD in the range of 1.736–1.985. Compared with PLS models, LS-SVM models performed better, with R^2^_p_ in the range of 0.848–0.858 and RPD in the range of 2.181–2.345. In all the models for L* prediction, the similar results obtained by LS-SVM model using the characteristic wavelengths selected by CARS and LS-SVM model based on full spectra were generally good. The R^2^_c_, R^2^_cv_, R^2^_p_ and RPD of these two models both exceeding 0.93, 0.81, 0.85 and 2.3, which illustrated the great performance of the model. Besides, standard deviation of the predicted values of the calibration set, validation set, and prediction set obtained by LS-SVM models were close to standard deviation of the measured values.

In the prediction of a*, R^2^_c_, R^2^_cv_, R^2^_p_ of all models exceeded 0.9 and RPD exceeded 3.9, exhibiting excellent performance. In addition, standard deviation of the predicted values obtained by all models and that of the measured values was quite close. The overall performance of LS-SVM models was better than that of PLS models. All the LS-SVM models achieved good results, with R^2^_c_, R^2^_cv_, R^2^_p_ and RPD were over 0.96, 0.94, 0.95 and 4.6. The results indicated that the model could effectively perform the prediction of the a* value of fresh-cut potato tuber slices.

For b* prediction, R^2^_c_, R^2^_cv_, R^2^_p_ of all models were greater than 0.85. LS-SVM models outperformed PLS models. The performance of the LS-SVM model based on characteristic wavelengths selected by CARS were similar to the LS-SVM model based on characteristic wavelengths selected by SPA. LS-SVM model established by full spectra yielded the best results. The R^2^_c_, R^2^_cv_, R^2^_p_ and RPD were up to 0.962, 0.909, 0.924 and 3.560 respectively.

As for the prediction of BI, PLS and LS-SVM models both showed good performance, R^2^_c_, R^2^_cv_, R^2^_p_ of all models exceeded 0.86, and the RPD range was 2.920–4.047. Both in PLS model or LS-SVM model, standard deviation of the predicted values of prediction set obtained by these two models were similar to standard deviation of the measured values. LS-SVM model built with full spectra performed best, with R^2^_c_, R^2^_cv_, R^2^_p_ and RPD were up to 0.958, 0.924, 0.940 and 4.047, indicating that the robustness of LS-SVM model based the full spectra.

For the prediction of L*/b*, R^2^_c_, R^2^_cv_, R^2^_p_ of most models exceeded 0.9, and RPD yielded 2.6. Among all the models for L*/b* prediction, the performance of the LS-SVM model based on the characteristic wavelengths selected by SPA and LS-SVM model based on full spectra were generally good, with R^2^_c_, R^2^_cv_, R^2^_p_ and RPD over 0.95, 0.91, 0.94 and 4.0 respectively. Besides, standard deviation of the predicted values of the calibration set and validation set obtained by these models were similar, and both of them were similar to standard deviation of the measured values. These results indicated that multivariate analysis of PLS and LS-SVM were conducive to characterize the color of fresh-cut potato tuber slices effectively.

In addition, it can be observed that the number of characteristic wavelengths selected by SPA and CARS was different, and the performance of the model established using the full spectra and the characteristic wavelengths showed slight difference. With SPA applied, the variable input was reduced 93.8–95.9% of the full spectra. Similarly, the variable input was reduced 88.4–93.5% of the full spectra by CARS. The performances of the PLS models and the LS-SVM models established by the characteristic wavelengths were comparable to or better than models based on the full spectra, which verified the effectiveness of the variables utilization by simplifying the input. Wavelengths selection contributed to avoiding the interference caused by redundant information and noise. Moreover, most models that utilized the characteristic wavelengths selected by CARS outperformed models that used wavelengths selected by SPA. In the prediction of a*, compared with the RPD (3.949) of the SPA-PLS model, the RPD of CARS-PLS model was significantly improved to 4.428. The same phenomenon was also observed in the L*/b* prediction.

In terms of model selection, all the models built by LS-SVM for all color index prediction were superior to the corresponding models established by PLS. In short, the nonlinear LS-SVM models performed better than the linear PLS models. This might be due to the outstanding processing ability of LS-SVM in linear and nonlinear problems, which contributed to not only handling the linear relationship between color index and spectra, but also better revealing the nonlinear relationship between the two. Therefore, LS-SVM was more suitable for the prediction of color index in fresh-cut potato tuber slices.

#### 3.3.2. Regression Models for Water Content Prediction

Table 3 shows the results of models for water content prediction. Overall, the model for water content prediction performed less satisfactorily, with R^2^_c_, R^2^_p_ and RPD ranging from 0.751–0.825, 0.718–0.794 and 1.675–2.018, respectively. The model performance of LS-SVM was significantly better than PLS, RPD was improved from 1.700 of CARS-PLS model to 1.978 of CARS-LS-SVM. In the similar study on the water content prediction of purple-fleshed sweet potato tuber slices [23], both R^2^_c_ and R^2^_p_ of PLS model reached 0.9, which were better than the prediction model in this study. The possible reason was that Sun et al. [24] explored the water content during drying process, and the gradient of water content was large. While we predicted the water content of the samples under room temperature storage conditions, the difference in water content between samples was relatively small. Besides, the number of characteristic wavelengths selected by SPA and CARS was different. The variable input was reduced to 5.4–5.9% of the full spectra by wavelength selection. The performance of the models established based on the characteristic wavelengths selected by CARS and SPA were comparable to or better than models based on the full spectra model based on the full spectra. In addition, standard deviation of the predicted values of the calibration set, validation set, and prediction set obtained by all models were close to that of the measured values.

Among the wavelengths selected by SPA and CARS (shown in Table S1 in Supplementary file), the wavelengths between 477–740 nm could be related to color information [36]. The selected wavelengths around 760 nm could be attributed to the absorption by O-H bonds [37]. The selected wavelengths near 840 nm may be attributed to the combinations of C-H stretching and C-H bending vibrations [38]. The selected wavelengths in the range from 880 nm to 890 nm could be attributed to the third overtone of C-H stretching [38]. In addition, the selected wavelengths near 906 nm and 928 nm could also be attributed to the third overtone of C-H stretching [38].

The quality change of potato during storage is a complicated issue. In addition to color index and water content, microorganisms are also one of the main factors affecting its quality. Studies have shown that with the extension of storage time, the microbial growth shows an increasing trend [39,40]. More work needed to be done to further study the effect of microbial growth on the quality during storage of potato tuber slices.

### 3.4. Visualization

The above results revealed that the color and water content of fresh-cut potato tuber slices could be evaluated quickly and effectively by HSI with the aid of multivariate analysis. In most cases, the performance of LS-SVM model based on full spectra was superior to other models. Therefore, LS-SVM model based on full spectra was utilized for visualization of the color index and water content of fresh-cut potato tuber slices. First, wavelet transform and area normalization preprocessing were performed on each pixel of the ROI, and then input the preprocessed spectra into the trained LS-SVM model, finally the color and water distribution of the entire sample were visually displayed.

#### 3.4.1. Color Visualization

**Figure 2:**
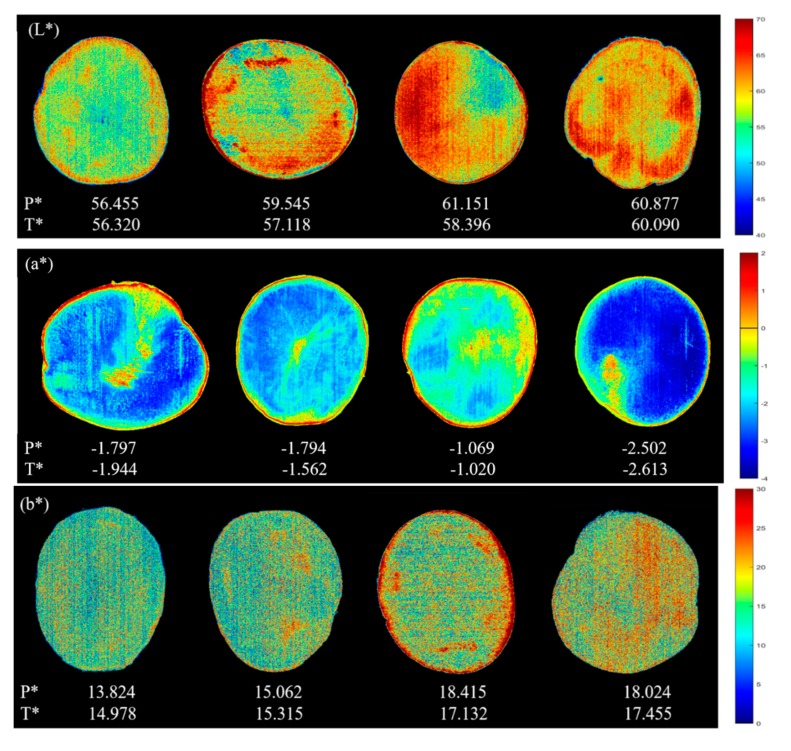

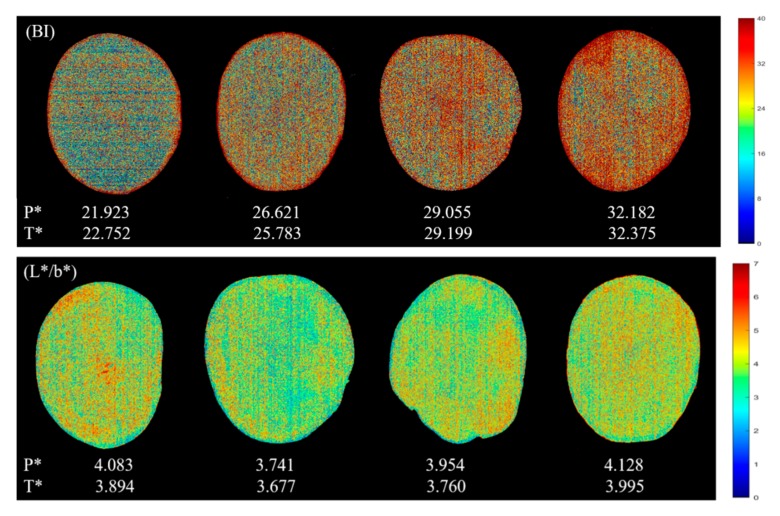
Pixel-wise prediction maps developed on the CARS-LS-SVM models for color visualization (P* is the predicted average value of all pixels; T* is the actual value measured by the chromatic aberration method).

The results of pixel-by-pixel prediction of the color distribution of fresh-cut potato tuber slices by the LS-SVM model are shown in Figure 2. The potato tuber slices were successfully separated from the background by means of multiple regression analysis, and the color bar corresponds to the continuous value of each color index within a certain range. Each pixel has a different color value from others. For fresh-cut potato tuber slices with higher values, more red pixels could be observed, while lower values correspond to more green pixels and blue pixels.

For the prediction of L*, b*, BI and L*/b*, although the values of each pixel are different, it presents a relatively uniform distribution in the whole ROI area. However, it can be seen from Figure 2 (a*) that the part near the middle of the potato sample is found to be darker, corresponding to a higher redness, which is mainly related to the location of browning. In addition, the main purpose of the chromatic aberration method commonly used is to evaluate the overall characteristics of the sample. Though calculating the average of the color values corresponding to each pixel may lose some spatial information, it is more feasible in practical application. In Figure 2, the P* value is the predicted average value of all pixels and the T* value is the actual value measured by the chromatic aberration method. According to the Figure 2 (L*), the values of L* measured by the chromatic aberration method were 56.320, 57.118, 58.396 and 60.090, respectively, which were similar with the average values of all pixels predicted by the LS-SVM model based on full spectra (56.455, 59.545, 61.151 and 60.877). The same phenomena could also be seen from the prediction of other color indicators (a*, b*, BI, L*/b*) in Figure 2, reflecting the validity and accuracy of LS-SVM models based on full spectra. As for BI, the higher the browning degree of the samples, the higher the BI value, which directly reflect the freshness of the fresh-cut potato tuber slices to some extent. Conversely, a higher L*/b* value indicates a higher brightness, a lower yellowness and a lower browning degree of potato tuber slices. The object oriented color prediction map based on LS-SVM model built with full spectra shows great potential to visualize color attributes, which will greatly facilitate the real-time nondestructive measurement in color of agricultural products.

#### 3.4.2. Water Content Visualization

**Figure 3:**
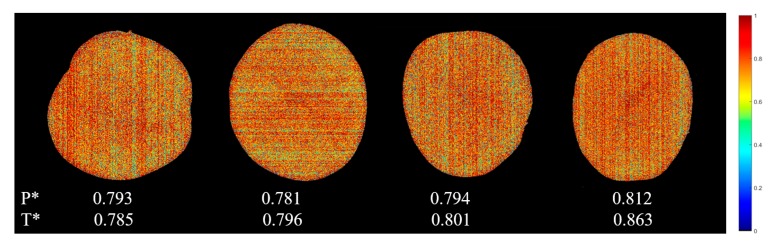
Pixel-wise prediction maps developed on the CARS-LS-SVM models for water content visualization (P* is the predicted average value of all pixels; T* is the actual value measured by the chromatic aberration method).

Visualization of water content distribution helps to quickly evaluate the water content of fresh-cut potato tuber slices. In this study, the LS-SVM model based on full spectra was used to calculate the predicted values of each pixel in hyperspectral images of fresh-cut potato tuber slices. Figure 3 shows the prediction map of water content distribution of four randomly selected samples. It can be seen from Figure 3 that the red pixels correspond to the tissues with higher water content, while the green and blue pixels indicate lower water content. Calculating the average values predicted of all the pixels is a good way to know the overall water content of the sample, which is usually the main concern in practical applications, rather than the specific location of the water distribution. The measured values of water content in the four samples were 0.785, 0.796, 0.801 and 0.863, respectively, they were quite similar to the average predicted values (0.793, 0.781, 0.794 and 0.812), with a minimum error of only 0.87% and a maximum error of 5.91%. It is shown that the water content of fresh-cut potato tuber slices can be predicted accurately based on the pixel information provided by HSI. The method has a great potential to detect the water content of potato tuber slices in real time, avoid the irreversible damage caused by traditional chemical methods and improve the detection efficiency and accuracy.

## 4. Conclusions

In this study, hyperspectral imaging system within the wavelength range 400–1000 nm coupled with multivariate analysis was applied for color index (L*, a*, b*, BI, L*/b*) and water content prediction of fresh-cut potato tuber slices. SPA and CARS were used to extract characteristic wavelengths, PLS and LS-SVM were used to establish regression models. Overall, the LSSVM models performed better than the PLS models. For color prediction, R^2^_c_ and R^2^_p_ of all the LSSVM models established for the five color indicators L*, a*, b*, BI, L*/b* were exceeding 0.90 and 0.84, respectively. All these results showed satisfactory performance of the models. For water content prediction, R^2^_c_, R^2^_p_, and RPD of the LSSVM models were over 0.80, 0.77 and 1.9, which was good but still need to be improved. LS-SVM models based on full spectra were used to visualize the spatial distribution of color and water content in fresh-cut potato tuber slices by means of pseudo-color imaging since LS-SVM models based on full spectra had the best performance in most cases. The good performance of hyperspectral imaging assisted by multivariate analysis demonstrated its effectiveness for color and water content prediction of fresh-cut potato tuber slices. In future studies, we will try to establish prediction models by deep learning methods to improve the performance of the models, and continue to explore the change of color and water content of fresh-cut potato tuber slices during shelf storage, in order to provide some references for relevant researches.

## Figures and Tables

**Table 1:** Statistical information of potato tuber slices in the calibration and prediction sets.

Indicator	Sample Set	Number	Range	Mean	Standard Deviation
L*	Cal ^a^	156	43.794–64.738	57.548	3.328
	Pre ^a^	78	45.497–64.681	57.561	3.301
a*	Cal	156	−3.096–+2.050	−1.277	1.308
	Pre	78	−3.045–+1.886	−1.278	1.31
b*	Cal	156	11.247–20.681	15.703	1.98
	Pre	78	11.581–20.567	15.704	1.977
BI	Cal	156	22.720–37.097	29.106	3.343
	Pre	78	22.752–36.278	29.104	3.357
L*/b*	Cal	156	2.921–4.401	3.696	0.315
	Pre	78	3.011–4.248	3.696	0.312
water content	Cal	156	0.753–0.879	0.811	0.0209
	Pre	78	0.758–0.876	0.811	0.021

a: Cal represents the calibration set, Pre represents the prediction set.

**Table 2:** Prediction results of L*, a*, b*, BI, L*/b* value by partial least squares (PLS) and least squares support vector machine (LS-SVM) models using full spectra, wavelengths selected by successive projections algorithm (SPA) and competitive adaptive reweighted sampling (CARS).

Models	Data Type	N.V. ^b^	Calibration			Validation			Prediction			
			R^2^_c_	RMSEC	SD_C_	R^2^_cv_	RMSECV	SD_CV_	R^2^_p_	RMSEP	SD_P_	RPD
L* value prediction												
PLS	Full	370	0.841	1.324	3.051	0.816	1.562	2.894	0.738	1.710	3.007	1.758
	SPA	23	0.838	1.333	3.047	0.802	1.622	2.717	0.736	1.723	2.993	1.736
	CARS	43	0.907	1.013	3.169	0.870	1.316	2.848	0.801	1.470	2.919	1.985
LSSVM	Full	370	0.938	0.827	3.174	0.814	1.437	3.126	0.858	1.298	3.010	2.319
	SPA	23	0.937	0.832	3.177	0.828	1.377	3.116	0.848	1.336	2.913	2.181
	CARS	43	0.932	0.865	3.165	0.834	1.353	3.089	0.851	1.305	3.061	2.345
a* value prediction												
PLS	Full	370	0.943	0.312	1.270	0.947	0.335	1.247	0.945	0.312	1.272	4.078
	SPA	15	0.928	0.351	1.260	0.931	0.381	1.255	0.941	0.318	1.255	3.949
	CARS	24	0.946	0.304	1.272	0.949	0.326	1.251	0.954	0.283	1.254	4.428
LSSVM	Full	370	0.976	0.201	1.281	0.949	0.294	1.276	0.956	0.274	1.289	4.704
	SPA	15	0.964	0.248	1.277	0.950	0.290	1.275	0.957	0.271	1.284	4.731
	CARS	24	0.966	0.239	1.281	0.950	0.292	1.279	0.957	0.272	1.275	4.686
b* value prediction												
PLS	Full	370	0.887	0.663	1.865	0.858	0.825	1.674	0.881	0.689	1.949	2.827
	SPA	21	0.899	0.628	1.877	0.862	0.816	1.695	0.887	0.679	1.982	2.918
	CARS	24	0.929	0.526	1.908	0.910	0.658	1.839	0.900	0.623	1.874	3.008
LSSVM	Full	370	0.962	0.383	1.930	0.909	0.597	1.938	0.924	0.546	1.942	3.560
	SPA	21	0.941	0.481	1.913	0.912	0.587	1.908	0.922	0.556	1.923	3.461
	CARS	24	0.959	0.399	1.927	0.909	0.597	1.920	0.924	0.548	1.895	3.457
BI value prediction												
PLS	Full	370	0.911	0.993	3.191	0.896	1.197	3.068	0.898	1.083	3.364	3.107
	SPA	17	0.887	1.121	3.148	0.862	1.379	3.030	0.887	1.141	3.333	2.920
	CARS	25	0.902	1.045	3.174	0.884	1.263	3.034	0.890	1.125	3.351	2.978
LSSVM	Full	370	0.958	0.685	3.248	0.924	0.922	3.231	0.940	0.823	3.328	4.047
	SPA	17	0.950	0.742	3.242	0.923	0.924	3.243	0.932	0.875	3.353	3.831
	CARS	25	0.958	0.686	3.256	0.932	0.869	3.244	0.929	0.899	3.297	3.669
L*/b* value prediction												
PLS	Full	370	0.904	0.097	0.299	0.885	0.118	0.289	0.872	0.114	0.300	2.634
	SPA	18	0.915	0.092	0.301	0.905	0.107	0.289	0.883	0.111	0.299	2.706
	CARS	30	0.938	0.078	0.305	0.928	0.093	0.294	0.929	0.087	0.296	3.390
LSSVM	Full	370	0.957	0.065	0.305	0.919	0.089	0.305	0.947	0.073	0.292	4.023
	SPA	18	0.954	0.068	0.305	0.922	0.088	0.305	0.948	0.072	0.293	4.093
	CARS	30	0.948	0.072	0.304	0.927	0.085	0.304	0.940	0.078	0.299	3.847

b: N.V. is the number of variables. SD_C_, SD_CV_, SD_P_: Standard deviation of the predicted values of the calibration set, validation set, and prediction set.

**Table 3:** Prediction results of water content by PLS and LS-SVM models using full spectra, wavelengths selected by SPA and CARS.

Models	Data Type	N.V. ^b^	Calibration			Validation			Prediction			
			R^2^_c_	RMSEC	SD_C_	R^2^_cv_	RMSECV	SD_CV_	R^2^_p_	RMSEP	SD_P_	RPD
PLS	Full	370	0.777	0.010	0.018	0.620	0.014	0.018	0.718	0.011	0.020	1.781
	SPA	20	0.751	0.010	0.018	0.624	0.014	0.017	0.719	0.011	0.019	1.675
	CARS	22	0.788	0.010	0.019	0.692	0.013	0.017	0.721	0.011	0.019	1.700
LSSVM	Full	370	0.812	0.009	0.018	0.692	0.012	0.018	0.778	0.010	0.020	2.006
	SPA	20	0.803	0.009	0.018	0.653	0.012	0.019	0.794	0.010	0.019	2.018
	CARS	22	0.825	0.009	0.018	0.713	0.011	0.019	0.791	0.010	0.019	1.978

b: N.V. is the number of variables. SD_C_, SD_CV_, SD_P_: Standard deviation of the predicted values of the calibration set, validation set, and prediction set.

## Supplementary Materials

The following are available online at https://www.mdpi.com/2304-8158/9/1/94/s1.
