# Supplementary material for: Rapid Screen of the Color and Water Content of Fresh-Cut Potato Tuber Slices Using Hyperspectral Imaging Coupled with Multivariate Analysis
Source: Foods. 2020 Jan 16;9(1):94. doi: 10.3390/foods9010094 (PMC7022740; doi:10.3390/foods9010094)
Supplement: Supplementary file 1 [file foods-09-00094-s001.pdf]

Table s1. Wavelength variables selected by SPA, CARS

Methods	Number	wavelengths (nm)
L^* value prediction		
SPA	23	490, 513, 532, 547, 562, 579, 605, 628, 636, 650, 658, 667, 673, 679, 698, 714, 720, 739, 751, 779, 804, 932, 947
CARS	43	528, 531, 532, 548, 549, 576, 580, 581, 583, 584, 591, 601, 605, 616, 667, 672, 681, 698, 715, 735, 737, 739, 755, 756, 761, 764, 778, 779, 783, 786, 787, 792, 801, 802, 804, 805, 841, 844, 845, 854, 857, 930, 940
a^* value prediction		
SPA	15	511, 531, 580, 606, 658, 668, 673, 679, 714, 720, 742, 751, 824, 920, 947
CARS	24	483, 489, 569, 586, 604, 666, 671, 672, 676, 700, 714, 784, 787, 788, 798, 802, 828, 852, 879, 880, 882, 899, 926, 946
b^* value prediction		
SPA	21	479, 485, 543, 586, 608, 626, 653, 659, 673, 679, 698, 709, 715, 739, 751, 773, 804, 823, 835, 920, 947
CARS	24	571, 579, 611, 624, 629, 742, 743, 753, 789, 791, 792, 793, 795, 804, 805, 806, 820, 823, 824, 832, 833, 845, 925, 930
BI value prediction		
SPA	17	609, 590, 578, 532, 820, 547, 519, 626, 918, 512, 833, 773, 484, 720, 688, 947, 667
CARS	25	495, 505, 521, 528, 550, 555, 576, 594, 595, 596, 610, 611, 613, 614, 626, 629, 630, 668, 674, 701, 715, 769, 770, 819, 891
L^*/b^* value prediction		
SPA	18	478, 485, 499, 513, 532, 548, 560, 608, 667, 678, 719, 729, 739, 773, 823, 873, 918, 947
CARS	30	526, 528, 537, 540, 549, 586, 589, 590, 610, 613, 614, 636, 698, 742, 756, 757, 792, 802, 804, 805, 820, 822, 823, 826, 831, 870, 884, 886, 888, 891
water content prediction		
SPA	20	485, 500, 521, 543, 578, 593, 623, 636, 652, 658, 667, 672, 679, 712, 752, 802, 899, 918, 937, 947
CARS	22	539, 547, 570, 584, 613, 629, 639, 643, 655, 704, 714, 752, 753, 780, 811, 859, 886, 895, 920, 929, 937, 944
